# Bixie Fenqing decoction in the treatment of chronic prostatitis: A systematic review and meta-analysis

**DOI:** 10.1097/MD.0000000000039558

**Published:** 2024-09-13

**Authors:** Hongying Li, Yanfang Yang, Menghua Shi, Lei Xu, Ying Huang, Zhaodi Hu, Guozheng Qin

**Affiliations:** a First Clinical Medical College, Yunnan University of Chinese Medicine, Kunming, China; b Department of Andrology, Reproductive Medicine, Yunnan Provincial Hospital of Chinese Medicine, The First Affiliated Hospital of Yunnan University of Chinese Medicine, Kunming, China.

**Keywords:** Bixie Fenqing decoction, chronic prostatitis, traditional Chinese medicine

## Abstract

**Background:**

Traditional Chinese medicine (TCM) posits that chronic prostatitis is associated with the accumulation of damp-heat pathogenic factors in the lower jiao. The Bixie Fenqing decoction (BFD) eliminates damp-heat pathogenic factors in the body, thereby alleviating inflammation and improving symptoms.

**Methods:**

Databases such as CNKI, WanFang, VIP, CBM, ClinicalKey, PubMed, Embase, and the Cochrane Library were searched. The search time ranged from the establishment of the database until March 30, 2024. RCTs that used BFD for chronic prostatitis were screened. The methodological quality of the studies was evaluated using the Cochrane Scoring System. Meta-analysis of outcome indicators was performed using RevMan 5.4 software, and Egger analysis of publication bias for the primary outcome indicators was conducted using Stata 16 software.

**Results:**

This analysis included 1104 patients. Meta-analysis showed that BFD significantly improved clinical efficacy in patients with chronic prostatitis, with a total effective rate (RR = 1.20, 95% CI: 1.13 to 1.26, *P* < .00001) and cure rate (RR = 1.52, 95% CI: 1.24 to 1.86, *P* < .00001). It significantly reduced the NIH-CPSI (National Institutes of Health-Chronic Prostatitis Symptom Index) scores, levels of inflammatory factors, white blood cell counts, and TCM syndrome scores in patients with chronic prostatitis. Specifically, the NIH-CPSI total scores (MD = −4.41, 95% CI: −5.27 to −3.55, *P* < .00001), NIH-CPSI pain scores (MD = −2.08, 95% CI: −2.93 to −1.23, *P* < .00001), NIH-CPSI urinary symptom scores (MD = −1.13, 95% CI: −1.69 to −0.57, *P* < .0001), NIH-CPSI quality of life scores (MD = −1.25, 95% CI: −1.76 to −0.75, *P* < .00001), levels of inflammatory factors TNF-α (MD = −11.18, 95% CI: −13.84 to −8.53, *P < *.00001) and IL-10 (MD = −20.60, 95% CI: −26.82 to −14.37, *P* < .00001) in prostatic fluid, white blood cell counts in prostatic fluid (MD = −2.91, 95% CI: −5.46 to −0.36, *P* = .03), and TCM syndrome scores (MD = −7.01, 95% CI: −8.13 to −5.90, *P* < .00001) were all significantly improved.

**Conclusion:**

BFD has a definite effect on the treatment of chronic prostatitis.

## 1. Introduction

Prostatitis, a syndrome with diverse clinical manifestations, is primarily characterized by abnormal urination symptoms and local pain or discomfort.^[1]^ According to the classification criteria established by the NIH, prostatitis can be categorized into 4 types, among which type III, known as chronic nonbacterial prostatitis or chronic pelvic pain syndrome, has an incidence rate as high as 90% to 95%.^[2]^ Chronic prostatitis, due to its intricate clinical symptoms and protracted course, not only significantly impacts patients’ physical health, but also has the potential to trigger psychological issues such as anxiety and depression.^[3]^

The Bixie Fenqing decoction, a classic prescription, was first documented in “Yang’s Family Collection.” It consists of an exquisite combination of 4 herbal ingredients: Fructus Alpiniae Oxyphyllae, *Dioscorea septemloba*, Radix Linderae, and *Acorus gramineus*. This formula was modified and developed by renowned physicians throughout history, including Danxi Zhu and Zhongling Cheng, eventually evolving into Cheng’s Bixie Fenqing decoction.

The updated formula comprises herbal ingredients, such as *D septemloba*, *Phellodendron chinense*, *A gramineus*, *Poria cocos*, *Atractylodes macrocephala*, Nelumbinis plumula, *Salvia miltiorrhiza*, and *Plantago asiatica*.^[4]^

“Bixie Fengqing Pill” is a traditional Chinese medicine compound preparation made based on “Bixie Fengqing Decoction.” Bixie Fenqing decoction (BFD), renowned for its remarkable efficacy in clearing heat and promoting diuresis, dispersing stasis, and relieving pain, is widely used to treat various symptoms of chronic prostatitis caused by damp-heat, including damp-heat descending, damp-heat stagnation, and damp-heat accumulation. Clinical practice has demonstrated that this formula effectively improves abnormal urination symptoms and significantly alleviates pain.

Several RCTs have scientifically evaluated the efficacy of Bixie Fenqing Yin in the treatment of chronic prostatitis. To more accurately assess its clinical efficacy and provide evidence-based medical support for clinical practice, this study aimed to conduct a systematic meta-analysis of these studies. Through in-depth analysis and aggregation of existing research data, we aspire to provide more scientific evidence for applying BFD in the treatment of chronic prostatitis.

## 2. Methods

### 2.1. Registration

This meta-analysis was performed according to the Preferred Reporting Items for Systematic Reviews and Meta-Analyses (PRISMA) guidelines^[5]^ and has been registered on PROSPERO (registration number CRD42024534471).

### 2.2. Systematic literature search

A systematic search was conducted in the CNKI, WanFang, VIP, CBM, ClinicalKey, PubMed, EMBASE, and Cochrane Library databases. All publications until March 30, 2024, will be searched without any restrictions on countries or article types. The Chinese search terms included Bixie Fenqing decoction, Bixie Fenqing pill, chronic prostatitis, chronic pelvic pain syndrome. The English search terms included chronic prostatitis, chronic pelvic pain syndrome, prostatitis, randomized controlled trial, and Bixie Fenqing decoction. Each search term was searched separately or in combination and a retrospective search was performed after screening the references of the included articles.

### 2.3. Inclusion criteria

RCTs that met the following criteria were included patients aged 18 to 67 who meet the relevant diagnostic criteria for chronic prostatitis; the full text of the study was available; intervention: the treatment group received BFD alone or in combination with other treatments, while the control group was treated with antibiotics, traditional Chinese patent medicines, western medicines, or conventional therapies. The baseline data of the 2 groups were comparable; outcome measures: the primary outcome measure was the total effective rate, and the secondary outcome measures included the cure rate, NIH-CPSI scores (total scores, pain scores, urinary symptom scores, and quality of life scores), inflammatory cytokines in prostatic fluid (TNF-α, IL-10), white blood cell counts in prostatic fluid, and traditional Chinese medicine (TCM) syndrome scores.

### 2.4. Exclusion criteria

Cannot obtain complete data of clinical research; repeated publications; articles without full text available; studies with incomplete, no available data, or incomplete data; animal experiments; review; systematic review; literature with low-quality evaluation; academic conference proceedings.

### 2.5. Study selection

Two researchers independently extracted all articles, and disagreements that emerged were resolved through group discussions. Two researchers read the titles and abstracts of all the studies, removed duplicate articles, and screened out articles that met the inclusion criteria.

### 2.6. Data collection

Two researchers independently extracted data from the final eligible articles, and the group decided on the disagreements that emerged. General information of the included literature, research methods, basic information of the research subjects, intervention measures of the treatment and control groups, and outcome indicators were extracted.

### 2.7. Risk of bias assessment

The Cochrane Collaboration tool to assess the risk of bias was used to assess the quality of the included studies. The methodological quality of the included studies was evaluated based on 7 aspects: random allocation method, allocation scheme concealment, blinding of subjects and investigators, blinding of outcome assessment, integrity of outcome data, selective reporting of study results, and other possible risks of bias. Disagreements that emerged were resolved through group discussions.

### 2.8. Statistical analysis

RevMan 5.4 (Informer Technologies, Inc., Los Angeles, CA, USA) was used to perform a meta-analysis of relevant data. Odds ratios were used as effect size indicators for dichotomous variables, and the mean and standard deviation were used for continuous variables. 95% confidence intervals (CI) were calculated. *P* < .05. *P* and *I*^2^ values were used to evaluate heterogeneity. There was no significant heterogeneity when *P* > .1 and *I*^2^ ≤ 50%, and the fixed-effects model was used. Otherwise, it indicated obvious heterogeneity. A random-effects model was used to analyze heterogeneity, and subgroup analysis or sensitivity analysis was used to explore heterogeneity when necessary.^[6]^ Egger analysis of publication bias of primary outcome measures was performed using the Stata software, version 15.1 (Stata Corporation, College Station, TX, USA).^[7]^ When *P* ≥ .05, the possibility of publication bias among studies was considered low.

## 3. Results

### 3.1. Literature search

**Figure 1.**
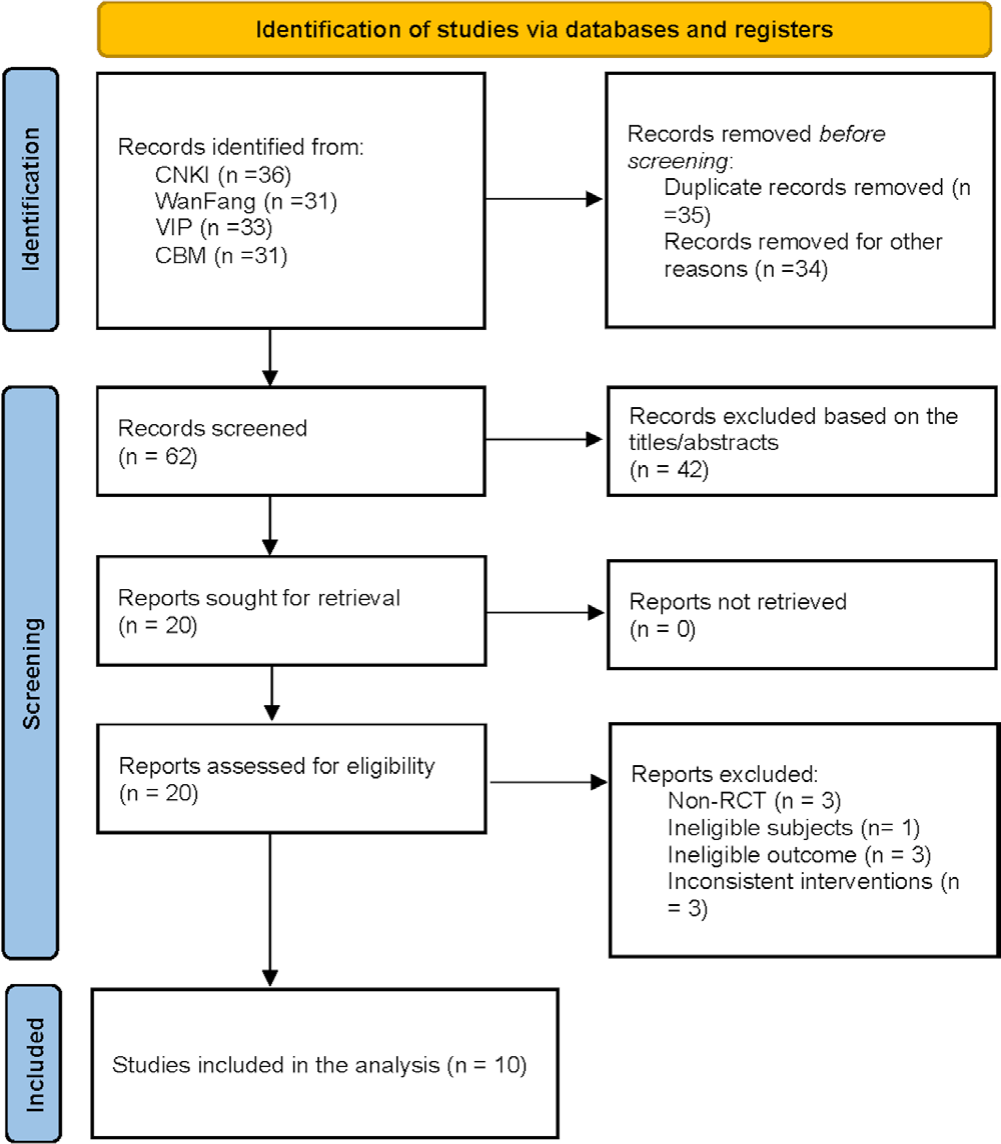
Flow chart of the literature screening process. CBM = China Biomedical Literature Database, CNKI = China National Knowledge Infrastructure, VIP = China Scientific Journal Database.

We retrieved 36 records from CNKI, 31 from WanFang, 33 from VIP, and 31 from CBM databases. After removing duplicate studies, we initially screened and deleted 42 articles. Following a review of the abstracts, 42 articles were excluded. After reading the full text, 10 articles were excluded for reasons such as non-RCTs (n = 3), ineligible subjects (n = 1), ineligible outcomes (n = 3), and inconsistent interventions (n = 3). Ultimately, 10 RCTs^[8–17]^ with 1104 patients were eligible and incorporated: 576 in the experimental group and 528 in the control group. The study screening process is shown in Figure 1, and the essential features of the eligible studies are presented in Table 1.

**Table 1:** Characteristics of included studies.

Study	Diagnosis	Sample size		Interventions		Trial duration (wk)	Outcomes
		T	C	T	C		
Peng 2015^[9]^	Chronic bacterial prostatitis	30	30	BFD	Antibiotics	4	①②⑩
Zhang 2015^[8]^	Chronic nonbacterial prostatitis	50	50	BFD	Prostat tablets	4	①②⑩
Lu 2016^[10]^	Chronic prostatitis	60	60	BFD + BP	Wengli tong capsules	8	①②⑩
Zhu 2017^[11]^	Prostatic pain	74	74	BFD	Flavone phenidate hydrochloride tablets	4	①②③④⑤⑥
Zhang 2018^[12]^	Type IIIA prostatiti	50	50	BFD + Antofloxacin tablets	Antofloxacin tablets	4	①②③⑨
Zhang 2020^[15]^	Type IIIA prostatiti	77	77	BFD + Clarithromycin tablets	Clarithromycin tablets	4	①②③④⑤⑥⑦⑨
Zheng 2020^[16]^	Chronic nonbacterial prostatitis	40	40	BFD	NingBi tai capsule	8	①②③④⑤⑥⑦⑧
Yu 2012^[13]^	Type IIIA prostatiti	78	30	BFP	Cerneton tablets	4	①②⑨
Wang 2019^[14]^	Chronic prostatitis	53	53	BFD + Levofloxacin hydrochloride capsules	Levofloxacin hydrochloride capsules	6	①②④⑤⑥⑦⑧⑨
He 2023^[17]^	Chronic prostatitis	64	64	BFD + Tamsulosin hydrochloride capsules	Tamsulosin hydrochloride capsules	4	①②⑥

Notes: T = Treatment group, C = Control group; BFD = Bixie Fenqing decoction, BFP = Bixie Fenqing pill, BP = Bazheng powder, ① = total effective rate, ② = cure rate; ③ = NIH-CPSI total scores, ④ = NIH-CPSI pain scores, ⑤ = NIH-CPSI urination symptom scores, ⑥ = NIH-CPSI quality of life scores, ⑦ = inflammatory factor TNF-α level in prostatic fluid, ⑧ = inflammatory factor IL-10 level in prostatic fluid, ⑨ = white blood cell counts in prostatic fluid, ⑩ = TCM syndrome scores.

### 3.2. Quality and risk of bias

**Figure 2.**
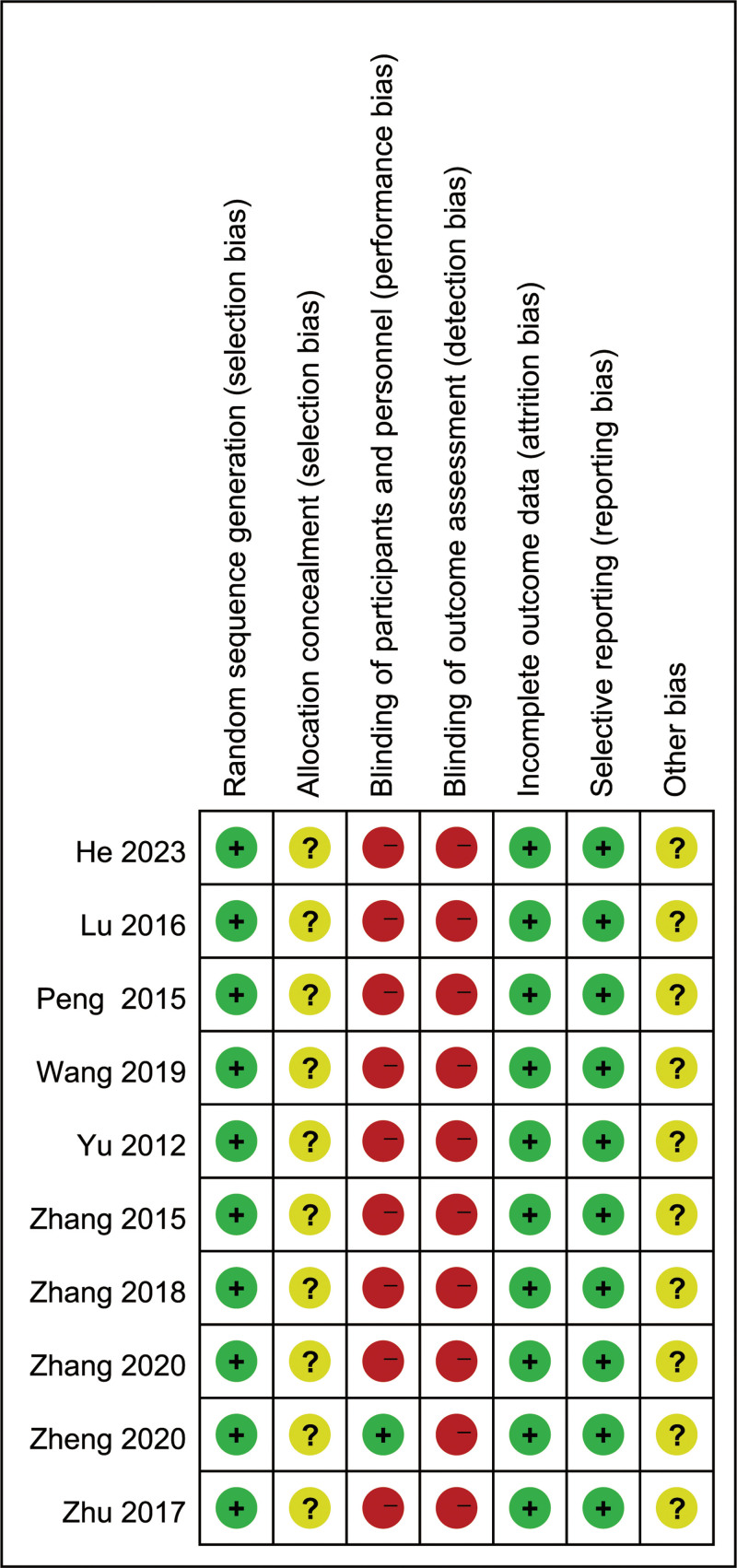
Graph summary of the risk of bias.

**Figure 3.**
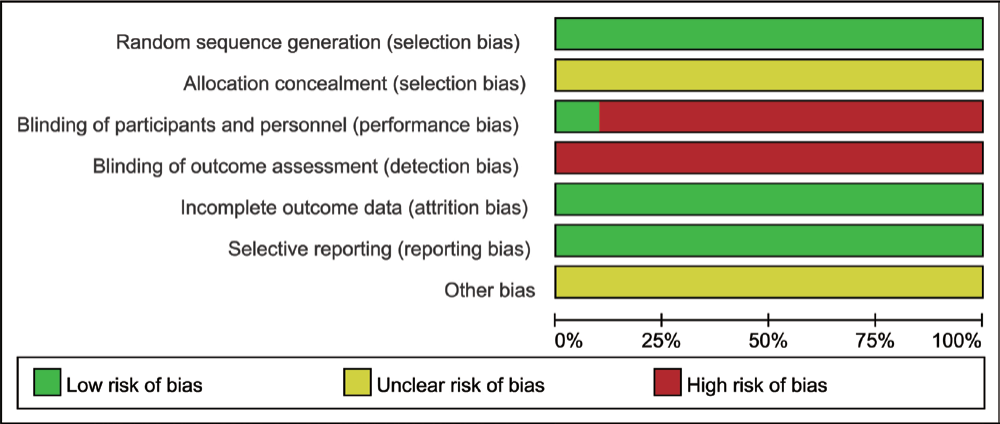
Assessment chart of the percentage risk of bias.

The 10 articles that were included all stated the use of a randomized controlled method, with 4 articles employing a random number table method, the remaining 6 did not specify the exact method used. Among these, 1 study adopted a double-blind method. None of the studies mentioned allocation concealment or blinding for outcome assessment, and all studies provided complete outcome data with no other potential bias risks (Figs. 2 and 3).

### 3.3. Systematic review and meta-analysis results

#### 3.3.1. Total effective rate

**Figure 4.**
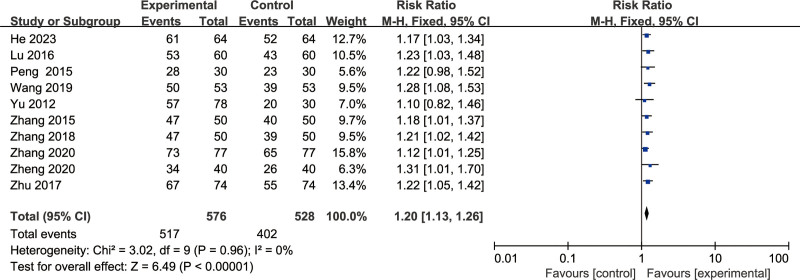
Forest plot of total effective rate.

Ten articles^[8–17]^ reported the total effective rates. Meta-analysis results showed that RR = 1.20, with a 95% CI of 1.13 to 1.26, and *P* < .00001, indicating that BFD can significantly improve the overall effective rate in the treatment group, and the difference was statistically significant (Fig. 4).

#### 3.3.2. Cure rate

**Figure 5.**
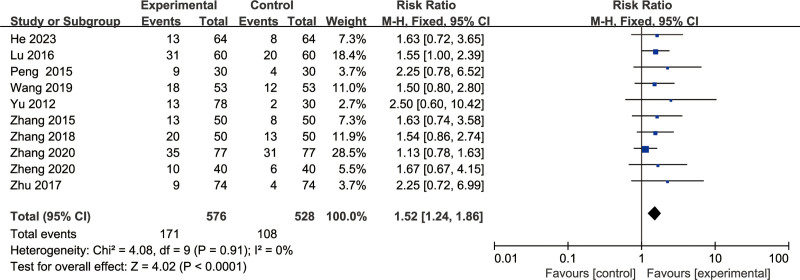
Forest plot of cure rate.

Ten articles^[8–17]^ reported cure rates. Meta-analysis results showed that RR = 1.52, 95% CI: 1.24 to 1.86, *P* < .00001, indicating that BFD can significantly improve the cure rate of the treatment group, and the difference is statistically significant (Fig. 5).

#### 3.3.3. NIH-CPSI scores

**Figure 6.**
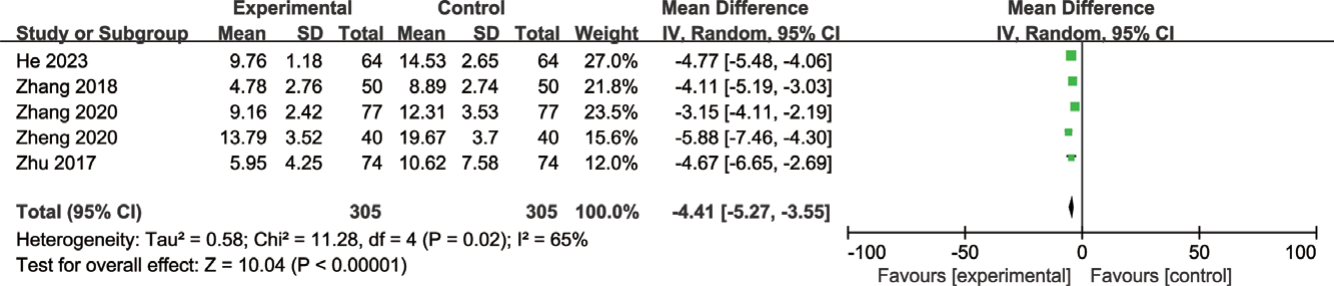
Forest plot of the total NIH-CPSI scores.

**Figure 7.**
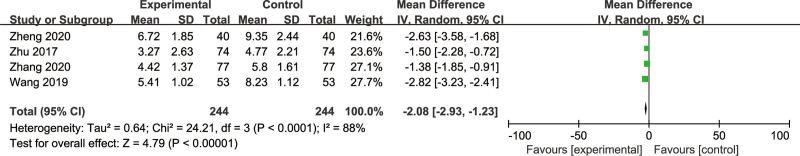
Forest plot of the NIH-CPSI pain scores.

**Figure 8.**
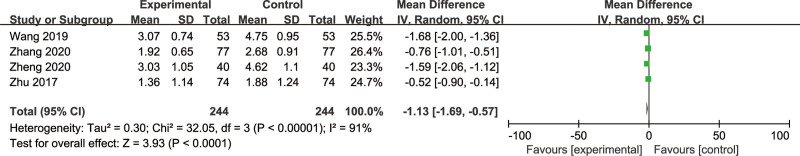
Forest plot of the NIH-CPSI micturition symptom scores.

**Figure 9.**
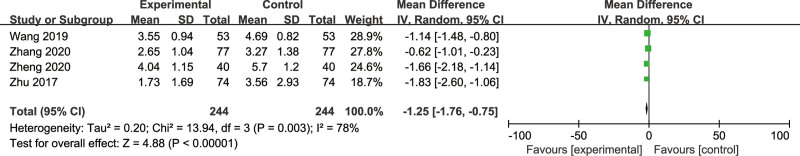
Forest plot of the NIH-CPSI quality of life scores.

The NIH-CPSI scores included the NIH-CPSI total, pain, urination symptom, and quality of life scores. Five articles reported^[11,12,15–17]^ the total NIH-CPSI scores, encompassing 610 cases. After heterogeneity testing, *P* = .02 and *I*^2^ = 65%, indicating heterogeneity among the 5 included articles. Therefore, a random-effects model was used in the meta-analysis. The meta-analysis results showed that MD = −4.41, 95% CI: −5.27 to −3.55, *P* < .00001 (Fig. 6). Four articles^[11,14–16]^ reported NIH-CPSI pain scores and included 488 cases. Heterogeneity testing revealed *P* < .0001 and *I*^2^ = 88%, indicating heterogeneity among the 4 articles. Hence, a random-effects model was employed for meta-analysis, which yielded MD = −2.08, 95% CI: −2.93 to −1.23, *P* < .00001 (Fig. 7). Four articles^[11,14–16]^ reported NIH-CPSI micturition symptom scores. Heterogeneity testing showed *P* < .00001 and *I*^2^ = 91%, indicating heterogeneity. Therefore, a random-effects model was used for the meta-analysis, MD = −1.13, 95% CI: −1.69 to −0.57, *P* < .0001 (Fig. 8). Four articles^[11,14–16]^ reported NIH-CPSI quality of life scores and contained 488 cases. Heterogeneity testing yielded *P* = .003 and *I*^2^ = 78%, suggesting heterogeneity. Thus, a random-effects model was applied for meta-analysis, which showed MD = −1.25, 95% CI: −1.76 to −0.75, *P* < .00001 (Fig. 9). In conclusion, BFD can significantly reduce patients’ NIH-CPSI total, pain, urination symptom, and quality of life scores, with statistically significant differences.

#### 3.3.4. Inflammatory cytokines in prostatic fluid

**Figure 10.**
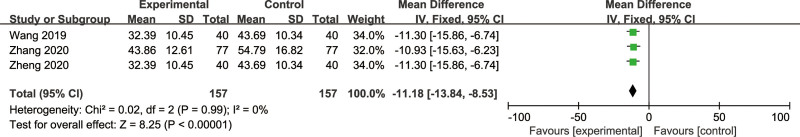
Forest plot of the inflammatory cytokine TNF-α level.

**Figure 11.**
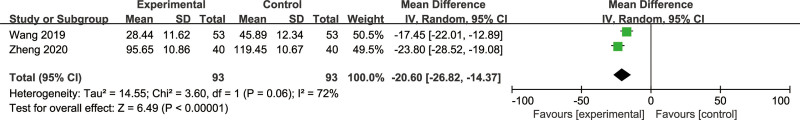
Forest plot of the inflammatory cytokine IL-10 level.

Inflammatory cytokines in the prostatic fluid include TNF-α and IL-10. Three articles^[14–16]^ reported the inflammatory cytokine TNF-α levels in the prostatic fluid and included 314 cases. Meta-analysis results showed: MD = −11.18, 95% CI: −13.84 to −8.53, *P* < .00001 (Fig. 10). Two articles^[14,16]^ reported the inflammatory cytokine IL-10 levels in prostatic fluid and included 186 cases. After heterogeneity testing, *P* = .06 and *I*^2^ = 72%, indicating heterogeneity between the 2 included articles. Therefore, a random-effects model was used in the meta-analysis. The meta-analysis results demonstrated MD = −20.60, 95% CI: −26.82 to −14.37, *P* < .00001 (Fig. 11). In conclusion, BFD can significantly reduce the levels of inflammatory cytokines TNF-α and IL-10 in the prostate, and the difference is statistically significant.

#### 3.3.5. White blood cell counts in prostatic fluid

**Figure 12.**
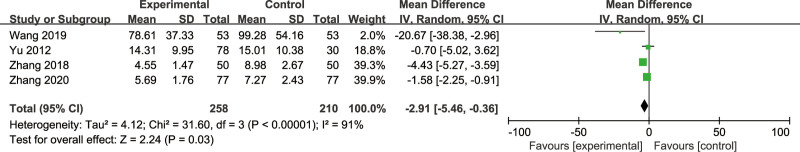
Forest plot of white blood cell counts.

Four articles^[12–15]^ reported white blood cell counts in prostatic fluid, including 468 cases. A random-effects model was adopted for meta-analysis after heterogeneity testing, with *P* < .00001 and *I*^2^ = 91%, indicating heterogeneity among the 4 studies. The results showed that MD = −2.91, 95% CI: −5.46 to −0.36, *P* = .03. This suggests that BFD can reduce white blood cell counts in the prostatic fluid, and the difference is statistically significant (Fig. 12).

#### 3.3.6. TCM syndrome scores

**Figure 13.**
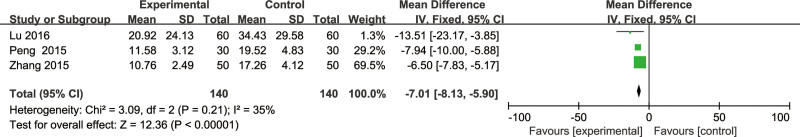
Forest plot of TCM syndrome scores.

Three articles reported TCM syndrome scores encompassing 280 patients. Meta-analysis results indicated that MD = −7.01, 95% CI: −8.13 to −5.90, *P* < .00001, suggesting that BFD can significantly reduce TCM syndrome scores, and the difference is statistically significant (Fig. 13).

### 3.4. Sensitivity analysis

To ensure the reliability of the results, we conducted a sensitivity analysis and found that the exclusion of each factor did not significantly affect the outcome indicators, thereby indicating the stability of the results.

### 3.5. Publication bias

Using the Stata 16 software, a publication bias analysis was conducted on the primary outcome measure. The results of Egger test indicated a low possibility of publication bias among studies examining overall effectiveness (*P* = .137), suggesting that the findings are relatively reliable.

## 4. Discussion

Chronic prostatitis, a prevalent and refractory disease in andrology, remains unclear in terms of its specific etiology and pathogenesis. Despite a lack of targeted treatment approaches, antibiotic therapy is widely used in clinical practice. According to TCM theory, this condition is categorized as “jing zhuo” (turbid essence), encompassing intricate pathologies that often involve a complex interplay of deficiency and excess syndromes. The underlying mechanism is primarily attributed to the downward flow of damp-heat facilitated by an insufficiency of vital qi, which sets the stage for disease onset. Additionally, qi stagnation and blood stasis persist throughout the disease progression. These 3 components collectively form the core pathology of the disease, highlighting the need for a multifaceted treatment approach that targets both the symptoms and their root causes. This approach primarily aims to clear heat and promote urination, supplemented by therapies designed to invigorate qi, activate blood circulation, disperse blood stasis, and alleviate pain. The integration of these treatment modalities underscores the significance of the TCM theory in managing this complex disease, emphasizing a comprehensive perspective that considers multiple factors simultaneously.

In the ancient Chinese medical textbook “Medical Insights: Red and White Turbidity,” it is documented that “there are 2 causes of turbidity: 1 arises from kidney deficiency leading to the leakage of depleted essence, and the other from damp-heat infiltrating the bladder. In cases of damp heat, it is crucial to regulate the spleen while treating dampness. For such situations, BFD can be chosen as the primary prescription.” Originally devised for the treatment of red and white turbidity, BFD, according to the TCM principle of “treating different diseases with the same therapy,” can also be effectively utilized in the treatment of damp-heat type chronic prostatitis, provided that the key pathogenesis of damp-heat descent is properly identified.

From the perspective of TCM, the efficacy of BFD in treating chronic prostatitis is primarily attributed to the exact composition of its formula. *Dioscorea septemloba*, with its bitter and neutral properties, primarily functions to eliminate dampness and resolve turbidity. *Plantago asiatica*, which is sweet and cold in nature, aids in draining damp heat from the bladder, and serves as a supporting herb. *Phellodendron chinense*, bitter and cold, can clear heat and dry dampness, drain fire, and tonify yin. When combined with *P asiatica*, it significantly potentiates the effect of clearing heat and promoting dampness excretion. Furthermore, Nelumbinis plumula, bitter and cold, clears heat from the heart and cools blood. *Salvia miltiorrhiza*, bitter and slightly cold, opens the heart orifice and clears blood heat. *Atractylodes macrocephala* and *P cocos* fortify the spleen and dry dampness, acting as assistant herbs in the formula. Finally, *A gramineus*, with its pungent, fragrant, bitter, and warm properties, harmonizes the heart and kidneys, assisting Bixie in amplifying the effect of resolving dampness and turbidity. Meticulous blending of these herbs contributes to the therapeutic outcomes of the formula.

From the perspective of modern pharmacology, various medicinal components of BFD have demonstrated significant pharmacological activity.

For instance, the chemical constituents of *D septemloba*, including steroids, diarylheptanes, and lignans, have been recognized for their antitumor, uric acid-lowering, and antifungal properties.^[18]^ The compounds α-asarone and β-asarone present in *A gramineus* modulate the expression of inflammatory factors and exhibit marked diuretic effects.^[19,20]^ Alkaloids found in *P chinense* possess anti-inflammatory and antibacterial actions, effectively mitigating prostatic protein exudation.^[21]^ Furthermore, the ethanol extract of *P asiatica*,^[22]^ pachymic acid from *P cocos*,^[23]^ atractylenolide from *A macrocephala*,^[24]^ tanshinone from *S. miltiorrhiza*,^[25]^ and components of Nelumbinis plumula^[26]^ also contribute to the treatment of chronic prostatitis through distinct mechanisms.

It is worth noting that although previous studies exploring the use of BFD in treating chronic prostatitis were largely anecdotal or based on individual experiences, this article presents more conclusive evidence through a meta-analysis of multiple RCTs. The findings reveal that BFD significantly improves not only the overall response rate and cure rate for patients but also reduces the NIH-CPSI scores and the levels of inflammatory cytokines in the prostatic fluid. Consequently, this leads to the alleviation of patients’ discomfort and urination abnormalities, thereby enhancing their quality of life. These results further substantiate the effectiveness of BFD in the treatment of chronic prostatitis.

In summary, BFD is an effective clinical treatment that can be used either alone or in combination with other medications to enhance its therapeutic effect. However, the findings of this study are subject to certain limitations, particularly the relatively small number of studies included, which may have affected the reliability of the results to some extent. In the future, we anticipate further refinement of this study by incorporating additional high-quality RCTs. It should also be noted that comprehensive observations on the safety of BFD treatment are lacking in the existing literature, and thus, safety assessments were not addressed in this analysis. Nonetheless, the efficacy of BFD in treating chronic prostatitis is evident and worthy of widespread clinical recommendation.

## 5. Conclusion

In conclusion, BFD could significantly improve the clinical efficacy in patients with chronic prostatitis, increasing the total effective and cure rates. Furthermore, it notably reduces the NIH-CPSI scores, levels of inflammatory cytokines in prostatic fluid, white blood cell counts in prostatic fluid, and TCM syndrome scores in patients with chronic prostatitis. Therefore, BFD deserves widespread clinical recommendations.

## Acknowledgments

We would like to thank all the authors for their contributions to this study.

## Author contributions

**Conceptualization:** Hongying Li.

**Data curation:** Yanfang Yang, Menghua Shi.

**Formal analysis:** Menghua Shi, Lei Xu.

**Funding acquisition:** Guozheng Qin.

**Methodology:** Yanfang Yang.

**Project administration:** Guozheng Qin.

**Software:** Lei Xu, Ying Huang.

**Validation:** Zhaodi Hu.

**Visualization:** Yanfang Yang.

**Writing – original draft:** Hongying Li.

**Writing – review & editing:** Hongying Li.
